# Suicide Related Phenotypes in a Bipolar Sample: Genetic Underpinnings

**DOI:** 10.3390/genes12101482

**Published:** 2021-09-23

**Authors:** Line K. M. Lybech, Marco Calabró, Silvana Briuglia, Antonio Drago, Concetta Crisafulli

**Affiliations:** 1 Unit for Psychiatric Research, Psychiatry, Aalborg University Hospital, DK-9100 Aalborg, Denmark; line.moeller@rn.dk; 2 Department of Biomedical and Dental Sciences and Morphofunctional Imaging, University of Messina, 98125 Messina, Italy; marco_3917w@hotmail.it (M.C.); sbriuglia@unime.it (S.B.)

**Keywords:** suicide, bipolar disorder, SNP, gene, molecular pathway analysis

## Abstract

Suicide in Bipolar Disorder (BD) is a relevant clinical concern. Genetics may shape the individual risk for suicide behavior in BD, together with known clinical factors. The lack of consistent replication in BD may be associated with its multigenetic component. In the present contribution we analyzed a sample of BD individuals (from STEP-BD database) to identify the genetic variants potentially associated with three different suicide-related phenotypes: (1) a feeling that the life was not worth living; (2) fantasies about committing a violent suicide; (3) previous attempted suicide. The sample under analysis included 1115 BD individuals. None of the SNPs reached genome-wide significance. However, a trend of association was evidenced for rs2767403, an intron variant of AOPEP gene, in association with phenotype #1 (*p* = 5.977 × 10^−6^). The molecular pathway analysis showed a significant enrichment in all the investigated phenotypes on pathways related to post synaptic signaling, neurotransmission and neurodevelopment. Further, NOTCH signaling or the γ-aminobutyric acid (GABA)-ergic signaling were found to be associated with specific suicide-related phenotypes. The present investigation contributes to the hypothesis that the genetic architecture of suicide behaviors in BD is related to alteration of entire pathways rather than single genes. In particular, our molecular pathway analysis points on some specific molecular events that could be the focus of further research in this field.

## 1. Introduction

Bipolar disorder (BD) is a group of recurrent affective disorders characterized by episodes of mania (a pathological elation of mood sometimes with psychotic symptoms), hypomania (a lesser form of mania) and/or depression (a pathological depressed mood sometimes with psychotic symptoms). BD definition has undergone major changes over the last decades. In this evolving classification system, one clinical phenotype remains of unchanged and vital relevance: suicide behavior. Suicide behavior is a prime concern in BD as BD patients hold the highest risk of suicide when compared with all other psychiatric subjects [1]: the expected lifespan in this disease is reduced by 8.5–16.7 years compared to the general population [2], and the higher suicide rates in BD are strictly implicated with this decrease [3]. Further, the high prevalence of BD, 0.3 to 1.5% in the general population, makes suicide risk an important concern in modern society [4,5] (Please refer to Appendix A for definitions of suicide related phenotypes and Appendix B for clinical risk factor along with preventive strategies for suicide behavior).

One-third up-to one-half of BD patients will attempt suicide at least once and 15–20% of BD individuals die from suicide. Suicide attempts in BD have a higher rate of success: 1 out 3–4 completers compared to the 1 out of 30 completers observed in the general population [6]. Genetics may be one of the factors contributing to the risk of suicide in BD, as reported by numerous adoption studies, twin studies and family studies. Data obtained evidenced that the rates of suicide attempts and completions are higher in individuals with a family history of attempters and/or completers (please refer to Table 1). Further, the genetic component for suicide seems to be partly shared with the inheritance of psychiatric disorders [7,8]: Indeed, recent GWAS analyses showed interesting correlation between suicide attempts and depressive symptoms, neuroticism, Schizophrenia, insomnia and major depressive disorder [9,10]. However, the genetic background behind suicide behaviors remains elusive: GWAS studies with polygenic risk scores have detected numerous SNPs potentially correlated with suicide [11,12,13]. The heritability calculated from these common variations only ranges between 4 and 4.6% [10,14], suggesting a complex biological background and a potential multifactorial origin behind this disease.

The high rates of suicide attempts and committed suicides with BD call for further research aiming at predict and hopefully reduce this behavior and ameliorate the devastating consequences for both relatives and the patients themselves. In this study we tried to investigate associations between suicidality and genetic variations in genotyped participants from the STEP-BD program (The Treatment Enhancement Program for Bipolar Disorder) [15] with a particular focus on biological pathways, which have a higher capacity of explaining complex phenotypes.

## 2. Materials and Methods

### 2.1. Clinical Sample

The sample under analysis was obtained from the NIMH genetics available Treatment Enhancement Program for Bipolar Disorder (STEP-BD). The STEP-BP remains one of the largest public investigations conducted so far for BD. It was located in the USA and initially enrolled 4361 participants distributed in 21 sites. Of them, only half gave consent for genetic analyses (please refer to Section 2.3). The trial included both naturalistic and randomized nested studies. Subjects had the opportunity to enter the randomized studies within the general trial once and re-enter the naturalistic design at exit. The study is described in details here [15].

### 2.2. Participants

Individuals included in the study were 18 years or older subjects with BD I or II. Diagnoses were confirmed by the Mini International Neuropsychiatric Interview (MINI) [35]. Moreover, a set of information was retrieved from the Affective Disorder Evaluation (ADE) set of questions prepared and focused on that specific study [15]. At the time of randomization all the subjects met criteria for acute depression at the MINI. Every patient received a standard care and follow-up, until accepting and meeting the criteria for being offered a randomized clinical treatment according to STEP-BD clinical states (SCSs) [15,36]. SCSs comprised: (1) the “acute depression SCS”; (2) the “refractory depression SCS”, which included patients that failed to respond to 12 months treatment or at least two trials in the past; and (3) the “relapse prevention pathway” in which the index episode (mania, hypomania or mixed) occurred in patients under either lithium or valproate treatment and with normal levels of Thyroid-stimulating hormone and creatinine [15,36].

### 2.3. Original Genetic Sample

About half of the original participants in the STEP-BD gave consent for the genetic analysis. From the original data (from the NIMH genetics database) there were 2453 people (1218 males, 1235 females), with a total genotype rate of 0.99 before quality control. Genotyping was performed using the Affymetrix GeneChip Human Mapping 500K Array Set (Affymetrix, part of Thermo Fisher Scientific, Waltham, MA, USA) by the Genetic Analysis Platform at the Broad Institute of Harvard and Massachusetts Institute of Technology. The link to the original study can be found at [15]. 372,193 variants were available in the original file before quality control. The Hapmap genome database b23 was instrumental for the imputation process. The sample was further filtered to exclude individuals with no information on the phenotypes under analysis. The final sample comprised 670 males and 485 females.

### 2.4. Outcomes

Three phenotypes were under analysis. Subjects that experienced (1) a feeling that the life was not worth living, AND/OR (2) fantasies about committing a violent suicide AND/OR (3) previous attempted suicide; were classified as “cases” or “controls” for main analysis: Individuals who had a score > 0 in one specific phenotype were considered cases for that phenotype. Individuals who had a score = 0 for a specific phenotype were considered controls for that specific phenotype. This analysis was performed with the aim of evidencing potential differences in the genetic background between the three phenotypes. These variables were chosen because they cover some relevant psychopathological aspects of suicide behavior, rather than the dichotomic attempted/completed suicide VS non suicide behavior. Additionally, an exploratory analysis which evaluated individuals with none of the described phenotypes as “controls” versus individuals with at least one of the described phenotypes as “cases” was performed. The aim of the exploratory analysis was to evidence the main genetic/biological differences between suicidal and non-suicidal bipolar patients. Only subjects with bipolar disorder were included in both analyses.

### 2.5. Clinical Covariates

Age, gender, ethnical background, marital status, living alone, education, kind of job and drug or alcohol abuse at the entry of the study, were the covariates included in the analysis. Variables are detailed in Table 1. Age, gender, ethnical background, marital status, living alone, kind of job and employment status and drug or alcohol abuse at the entry of the study were retrieved from the DF database and downloaded after permission from the NIMH genetics database. As for the ethnical background, the questionnaire included two questions, the first being “What is your primary race” and including answers as for example, “White or Caucasian” or “Black of African American”. The second was “Are you Hispanic or Latino?” BD classification type was excluded from covariates since the phenotype under analysis is associated with the depressive phase, which is similar in type I and type II and this classification is based mainly on manic phase.

### 2.6. Statistical Model and Flow of Analysis

All analyses were conducted in R [37], or in bash environment. Plink [38], gtool (https://www.well.ox.ac.uk/, (accessed on 1 April 2021)) and impute [39] were instrumental to perform the genetic analyses. The bash environment provided the basis for the use of plink, impute and gtool. It also allowed for an effective manipulation of phenotype data, in order to adapt them to the input requirements for the above-mentioned programs. The single nucleotide association analyses were conducted in Plink after the genotype was imputed (with gtool and impute), pruned and checked for quality control to standard defaults for this kind of analysis. Molecular pathway analyses were performed on R environment, using the result of the single nucleotide association analysis as input, and providing as output the molecular pathways found to be enriched in mutations associated with the phenotypes under analysis. Figures and tables were also created in R. R script is available on request.

### 2.7. Analysis of Clinical Data

The clinical phenotypes were created and their association with the clinical covariates tested with the appropriate statistical test (for example ANOVA or Chi2, or the correspondent nonparametric test) in order to protect the genetic analysis from clinical stratification factors. Covariates that were significantly associated with the outcomes under analysis were included in files containing binary variables. For example, if “living alone” was found to be associated with the phenotypes under analysis, the variable was commuted to a binary 1 or 2 variable where 1 was for example “married or living as married” and 2 contained every other clinical variation of the original variable. This process was instrumental to reduce the degree of freedom of the analysis, to avoid the risk of a NA (not assessable) result from the plink analysis (genetic association analysis). 

### 2.8. Analysis of Genetic Data

**Figure 1:**
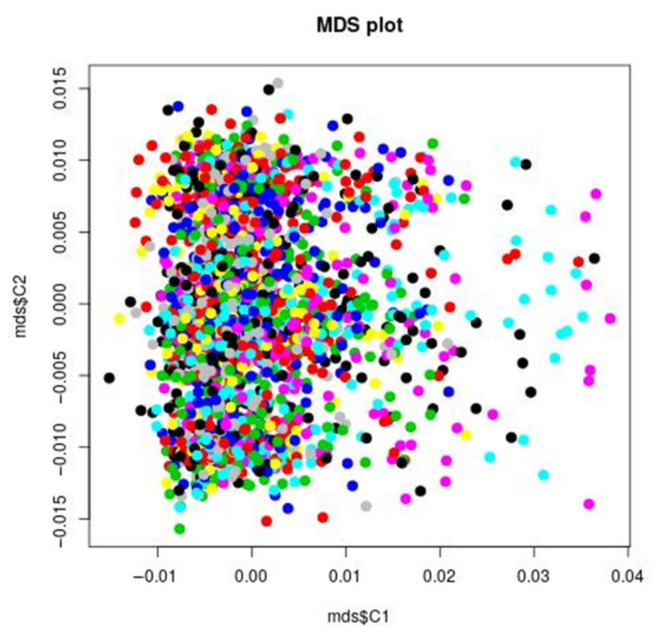
MDS plot: IBS clustering analysis of the genetic stratification factors. Increasing values of the first component do not correspond to visual significant increasing or decreasing values of the second component, suggesting that the covariance between the two components in the genetic is minimal. For example, to small and increasing values of the first component correspond both small and higher values of the second component. The points are concentrated in the left end of the figure, indicating a larger variance of the first component. In order to further test this visual impression, the plink permutation test for between group IBS differences confirmed that there was no significant group genetic differences (stratification factors) with respect to all the phenotypes under analysis. That does not necessarily mean, that there is no genetic stratification in the STEP-BD sample, but that this stratification is not of main significant interest when considering the phenotypes under analysis. The pairwise clustering based on IBS (identity by state) is useful for detecting pairs of individuals who look more different from each other than what is expected in a random, homogeneous sample. This method allows for identification of clusters of patients, that are more genetically similar to each other than they are similar to the rest of the sample. Such groups are identified by different colors in the figure.

The standard quality thresholds were applied to the original sample before pruning and imputing. Minor allele frequency was set at 0.05, genotype rate was set at 0.95, Hardy Weinberg Equilibrium was set at 0.00001. Pruning was set at the standard --indep 50, 5, 2, where 50 is the number of SNPs considered at every step, 5 is the number of SNPs to be shift at every step and 2 is the VIF threshold (1/ (1 − R^2^) where R^2^ is the multiple correlation coefficient). An R^2^ equals to 10 implies that two SNPs carry the same signal. An R^2^ equals to 1 implies that two SNPs are completely independent. The enrichment analysis was conducted using R software suite, through Bioconductor [40] and the package ReactomePA [41]. The ReactomePA (https://bioconductor.org/packages/release/bioc/html/ReactomePA.html, accessed on 1 April 2021) is a manually curated database that includes chemical reactions, biological processes and molecular pathways. To test for possible stratification factors associated with the phenotypes under analysis at plink test was performed under standard parameters for test the non-genetic identity of the samples (cases and controls, Permutation test for between group IBS differences where IBS stands for identical by state). Please refer to Figure 1 and Table 2. Single tests for association were generated for every SNP under a regression model. SNPs associated with the investigated phenotype were ranked according to the pValue of association. SNPs showing a significant (*p* < 0.05) association with the phenotypes under analysis were selected. It is of note, that this level of significance is not be used in a classic GWAS study because of the risk of false positives. This—or similar—classic level of significance was nevertheless chosen in the present as in previous studies [42,43,44,45,46] to identify all the possible significant associations throughout the genome. Moreover, the same level of significance for this kind of analysis is standard for the package in use. The risk for false positives was then controlled by correcting for multiple testing, a function that is embedded in the R package in use. In particular, Bonferroni correction (*p*.adjust) and False Discovery Rate correction (*q* value) were provided. The genes that harbored such variations were identified and investigated for enrichment. Enrichment in this context means, that the number of SNPs that are significantly associated with the phenotypes under analysis is larger than expected by chance. As a consequence, the genetic variations distributed in specific molecular pathways, those enriched, are shown to have a potential role in differentiating cases from controls. This approach takes into consideration the likely multigenetic nature of such complex phenotypes and provides more power for this kind of analysis. The analysis was conducted in a Linux system in Bash language; the computations were conducted through access at the Aalborg University superPC.

## 3. Results

The sample under analysis and the covariate analysis are described in Table 3. Gender was not included in the analysis because not associated with the phenotypes under analysis and because there was no evidence for major genetic stratification factors for the phenotypes under analysis. 

### 3.1. Main Analysis

**Figure 2:**
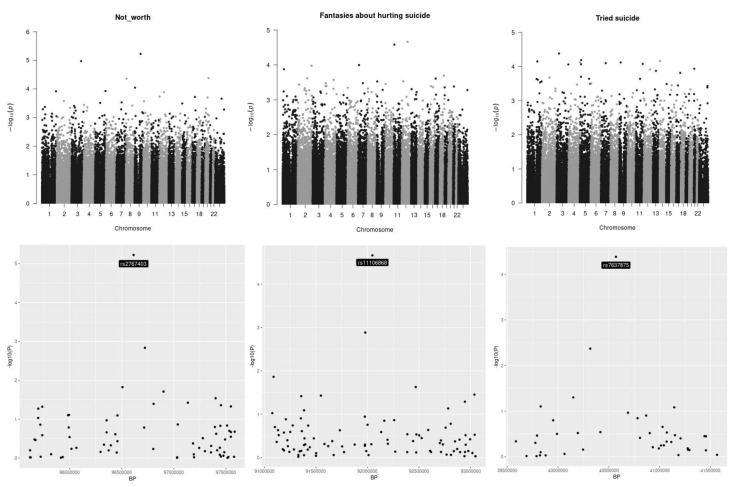
Manhattan plots. Manhattan plots for the three outcomes under analysis are presented in the top of the picture. At the bottom of the picture the genomic areas with the strongest associations are shown. The outcomes under analysis are, respectively, from left to right, “having a feeling of not worth”, “fantasies about a hurting suicide” and “tried suicide”. None of the single investigated SNP reached the genome-wide threshold.

**Figure 3:**
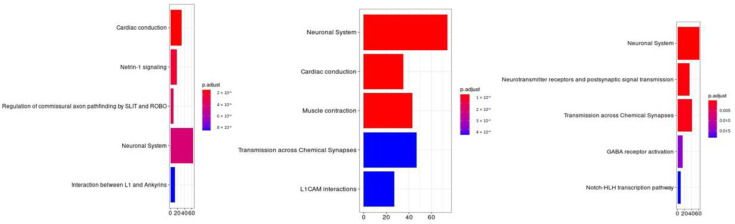
Molecular pathway analysis. The molecular pathways are listed in the Y axis. X axis describes the number of genes found to be enriched in association with, respectively, from left to right, “having a feeling of not worth”, “fantasies about a hurting suicide” and “tried suicide”.

Figure 1 reports the analysis of the genetic stratification factors. Figure 2 reports the result of the genome wide analysis. Figure 3 reports the result of the molecular pathway analysis. 957418 SNPs were available for the analysis after standard quality control and imputation. Briefly, no single variation reached genome wide significance in the GWAS analysis. The strongest association resulted for rs2767403 (C > G) (*p* = 5.977 × 10^−6^) in association with phenotype #1 (Not worth). Rs2767403 is an intron variant of the AOPEP (aminopeptidase O), a gene implicated in the angiotensin IV pathway. 

According to in silico analyses of the effects of rs2767403 on splicing, this mutation introduces a donor splice site within the *AOPEP* sequence, which can potentially alter the splicing pattern of *AOPEP* mRNA. Table 4 reports the results of the in silico analysis that was performed using Genomnis HSF software (https://www.genomnis.com/, accessed on 21 April 2021).

**Figure 4:**
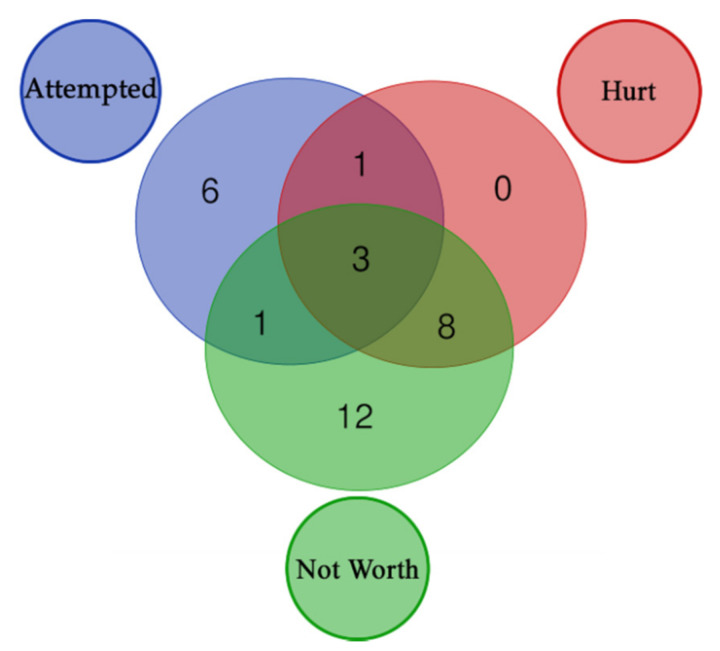
Distribution of enriched pathways for the 3 suicide classes. The figure reports the Venn Diagram of enriched pathways distribution for the 3 suicide classes. Attempted + Hurt + Not Worth (3): R-HSA-373752, R-HSA-112316, R-HSA-112315; Attempted + Hurt (1): R-HSA-112314; Attempted + Not Worth (1): R-HSA-428542; Hurt + Not Worth (8): R-HSA-397014, R-HSA-5173105, R-HSA-1474244, R-HSA-445095, R-HSA-5578775, R-HSA-5576891, R-HSA-373760, R-HSA-5576892; Attempted (6): R-HSA-421270, R-HSA-350054, R-HSA-446728, R-HSA-1500931, R-HSA-977443, R-HSA-418990; Not Worth (12): R-HSA-1650814, R-HSA-5083635, R-HSA-5173214, R-HSA-1474290, R-HSA-8948216, R-HSA-3906995, R-HSA-2022928, R-HSA-375165, R-HSA-3000178, R-HSA-983712, R-HSA-419037, R-HSA-936837.

**Figure 5:**
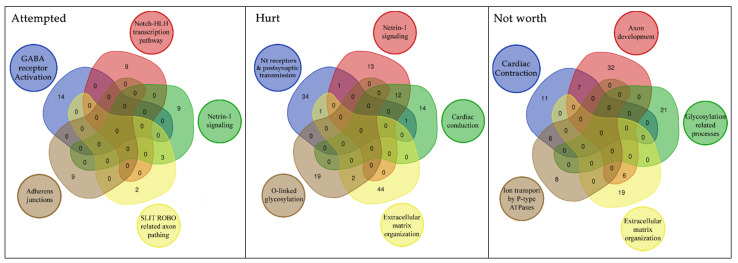
Overlap of the genetic structure associated with specific suicide related phenotypes in BD. This figure reports the unique pathways we found involved in the three suicide classes. Further we reported through a venn diagram the number of genes shared by each of these pathways.

The molecular pathways analysis reported a list of molecular pathways enriched in genetic variations associated with the phenotypes under analysis. Some shared molecular pathways enriched in variations associated with all the phenotypes under investigation were the pathways involved in post synaptic signaling, neurotransmission in general and neurodevelopment (axon). Finally, NOTCH signaling and the GABAergic signaling were found to be associated with specific suicidal behaviors (please refer to Table 5, Figure 4 and Figure 5).

### 3.2. Exploratory Analysis

**Figure 6:**
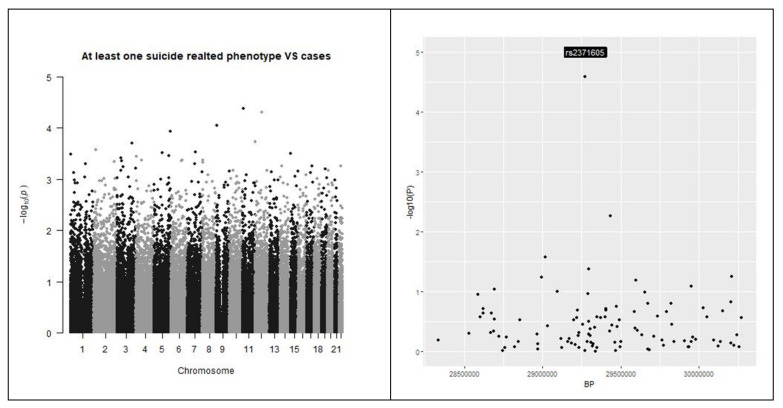
Manhattan plots. Left: Manhattan plots for the outcome under analysis were reported. Right: the genomic areas with the strongest associations with outcome are shown. The outcome under analysis is suicidal behavior versus controls. None of the single investigated SNP reached the genome-wide threshold.

**Figure 7:**
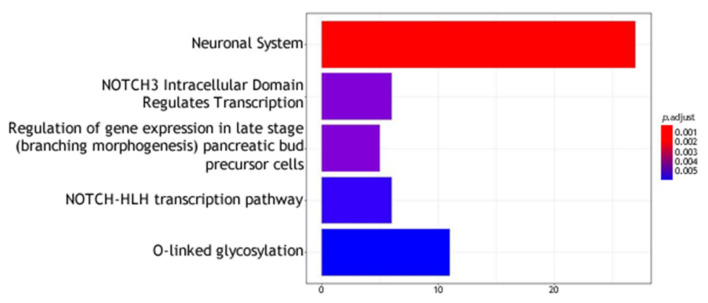
Molecular pathway analysis. The molecular pathways are listed in the Y axis. X axis describes the number of genes found to be enriched in the suicidal behavior versus controls analysis.

The result of the analyses on individuals reporting at least one of the investigated phenotypes (cases) against individuals reporting none (controls) showed no significant data in single SNPs analysis (please refer to Figure 6). The molecular pathways analysis reported a list of molecular pathways enriched in genetic variations associated with the phenotypes under analysis. Results are reported in Table 6 and Figure 7.

## 4. Discussion

Suicidal attempts are dramatic, frequent events in psychiatric clinical practice. Even though several studies have been carried out, there are no data on the biological factors influencing the risk of this event that can be used in the current clinical practice. In this paper we have focused on the potential genetic backbone which may be indicative of risk in BD.

### 4.1. Analysis of Single SNPs

In our study, no single DNA variation reached genome-wide significance, emphasiz ing that no gene or SNP, alone, has a high enough impact on suicide risk. Still, some insights could be obtained from the observed trend. The most significant association, with a *p* = 5.977 × 10^−6^, was related to an intronic variant (rs2767403) of the *AOPEP* gene. The other two SNPs with a low (but not significant) *p*-value (rs11106868 and rs7637875) were localized in intergenic regions as such no further analysis was conducted. Regarding the exploratory analysis, no SNPs reached the significant threshold and the most significant trend (rs2371605) was localized in an intergenic region. According to in silico analyses, rs2767403 introduces a donor splice site within the *AOPEP* sequence (Table 4).

Even though this prediction highlights the potential variability caused by rs2767403, the *AOPEP* gene encodes a metallo protease linked to the renin-angiotensin system [47]. In particular, it is able to cleave angiotensin III (but not I and II) to generate Angiotensin IV. It is mainly expressed in heart, but it can also be found, with lower concentrations, in brain. Overall, *AOPEP* RNA has poor tissue specificity and can be found in almost all tissue of the body. Unfortunately, except for its correlation with the renin-angiotensin system, little is known about its function. Of note, several studies consistently associated *AOPEP* with polycystic ovary syndrome (PCOS) [48,49,50,51,52,53]. 

Of interest, the *AOPEP* region also holds the miR-23b/27b/24 cluster [54]. This cluster is composed of three miRNA genes located within 14 intron of *C9orf3* (*AOPEP*) [54]. The precise mechanism of regulation of the miR-23b/27b/24 cluster expression is still not clear [54]. According to current knowledge on intronic miRNAs biogenesis, the pri-miR-23b/27b/24 cluster is potentially transcribed as part of the transcript of the *AOPEP* gene. Therefore, potential alterations of *AOPEP* maturation processes by rs2767403 can potentially interfere with the cluster expression.

The role of these miRNAs has not been fully elucidated, however studies examining the roles of miR-23b, miR-27b and miR-24-1 have demonstrated their multiple functions, ranging from metabolic disorders to proliferation and development disorders [55,56,57,58,59,60]. These miRNAs are highly expressed in vascularized tissues [60]. In addition, miR-27b was shown to target the NOTCH ligand Delta-like ligand 4 (Dll4) [55]. To note, the NOTCH pathway was enriched in our pathway analysis. Within their functions, this cluster, and in particular miR-23b and miR-27b, seem to exert an important control on neuronal apoptosis, modulating the expression of *Apaf-1* gene (in a murine model) [61]. Apaf-1 is a key apoptotic protein associated with neuronal apoptosis [62]. This protein levels are extremely low in adult brains. Conversely, the expression of miR-23-27 cluster is significantly higher in adults than in embryos. Even though its function in vivo remains elusive, this cluster likely regulates Apaf-1 expression, thus affecting the sensitivity of neurons to apoptosis during development. A potential alteration of this tight regulation system can lead to impaired neural nets (brain) development. Even though there is currently no evidence in literature that correlates this cluster and suicide, miRNAs expression can be closely related to neurophysiology and suicidal behavior [63,64]. 

### 4.2. Molecular Pathway Analysis

Regarding our pathway analysis, our data evidenced that specific molecular cascades are enriched in the above defined suicide classes (please refer to Table 5). The three classes of suicide (Attempted, Hurt and Not worth) shared some of these pathways, as also evidenced by exploratory analysis, while others were unique. In particular, the biological processes involved in post synaptic signaling, neurotransmission in general and neurodevelopment (axon) were enriched in all these classes and in the exploratory analysis (Neuronal System), although GABA signaling was only enriched in the Attempted class. In addition, NOTCH signaling and cell-cell communication pathway were found to be significantly enriched only in this class. The Not worth class was related to the biological processes involved in ion equilibrium, with extracellular matrix and glycosylation processes. Figure 4 reports a Venn diagram showing pathways distribution in the 3 classes. 

A further focus on the genes within each enriched pathway explains the significance of some apparently unrelated pathways. Indeed, as can be seen in the Venn diagrams (Figure 5), cardiac conduction related pathway shares 12 genes with Netrin-1 signaling (axon development related process). Common genes are the *SCN* genes, which encode for sodium voltage-gated channel subunits. They are important in both cardiac and brain function. In particular, the pathway associated with *SCN* genes seems to be involved in the biological processes of pain, especially in the development of inflammatory pain [65,66,67,68]. This could explain the link between these genes (and related pathways) and suicide risk, as pain is a robust predictor of suicidal desire [69].

In the Not Worth class, the pathway of cardiac function also shared its genetic background with the ion transport by P-type ATPases pathway. The genes shared by the two play a role in both cardiac function and neurotransmission related processes. Transmembrane ion transport by ATPases is closely related to the membrane potential. Alterations within this process likely alter neurotransmission processes. Interestingly, literature data associate ATPase activity coupled to the transport of ions across the cell membrane with suicide risk [70,71].

The associated pathways related to the neurotransmission processes (common in the three classes) and to GABA neurotransmission, specific for suicide attempters were deeply investigated in the literature. The data obtained showed that the perturbation of glutamatergic and GABAergic neurotransmission systems, which play roles in excitatory and inhibitory responses, respectively, contribute to the neurobiology of psychiatric disorders and have been associated with suicide [70,71,72,73,74,75,76,77,78,79]. The mechanisms by which such complex behaviors are not well understood, but likely involve the function of both ionotropic GABA_A_ and metabotropic GABA_B_ receptors (GABA_A_R and GABA_B_R) [79,80].

In addition, the Attempted class was associated with processes related to adherent junctions. The molecules involved in this cascade have been linked to anxiety and mood disorders [81], which represent substantial risk factors contributing to suicidal behavior [81].

Finally, NOTCH signaling was also associated with the class of suicide attempters. Recent studies have associated alterations of neuronal plasticity in specific brain areas with suicidal behavior [82]. In this context, the NOTCH signaling pathway plays a relevant role in neuronal plasticity as well as cell survival and migration [83], which are biological processes proven to be altered in suicide victims [84]. It also has been identified as critical regulator of neurogenesis and gliogenesis in the adult brain [85,86]. During the last decade, alterations in neurogenesis processes were identified in suicide victims [87]. The alteration of these processes is supported by the structural anomalies that can be observed in different brain areas of suicide attempters such as PFC and HIP [87,88,89]. The role of the NOTCH signaling pathway in neurogenesis strongly suggests its potential involvement in suicidal behavior.

The Hurt and Not Worth classes were found to be linked to less neuron-specific processes: Glycosylation and ExtraCellular Matrix (ECM) Organization. Even though, these biological cascades may not seem to be important for brain functioning, literature data has demonstrated their essential role for the physiological function of the brain [90]. Indeed, a large portion of the brain volume is made up of ECM and its interaction with local cells is essential for functions such as memory and learning [91]. ECM also has a prominent role in brain development, maturation of neural circuits and adult neuroplasticity (cell migration, axonal outgrowth and synaptogenesis) [92]. It also has important roles in neurotransmission and signal transduction since it influences the exchange of ions and neurotransmitters through extracellular space [92,93]. Proteins used to anchor cells to ECM, such as CD44, have been found to be expressed by several brain cells including neurons [94], astrocytes [95] and microglia [96]. Interestingly, these proteins required to organize ECM have been found to be related to suicidal behavior [97,98], likely influencing brain homeostasis through their immune control [90,99] and regulation of the blood-brain barrier permeability [90,100]. This multifactorial role of the ECM suggests that alterations in its organization could potentially lead to impaired brain function and, consequently, increasing the risk for neuropsychiatric and/or neurodegenerative diseases [90,101,102]. The role of ECM is strongly related to glycosylation. In the brain, the formation and structure of the ECM contains the hyaluronic polysaccharide, and a large variety of glycoproteins and proteoglycans [103]. The glycosylation process is tightly regulated as it is an enzymatic modification that is site and substrate specific [104]. Further glycosylation plays an important role in various cellular processes, from cell adhesion and pattern recognition [105] to more specific brain-related roles in controlling neurite sprouting and development [104,106,107]. This process may alter the synthesis of gangliosides, impairing the physiological function of the brain [108]. Even though no literature data exposed a precise causal correlation between altered glycosylation processes and suicidal behavior, this pathway is implicated in several disease states including psychiatric and neurodegenerative disorders [109,110,111,112,113,114,115,116].

## 5. Conclusions

Bipolar Disorder (BD) is a recurrent, frequent and devastating affective disorder. The current treatments mitigate the exacerbations of BD symptoms at the cost of side effects, including weight gain, together with a non-optimal efficacy. Moreover, suicide rates are higher among individuals with BD. Clinical interviews and treatment alliance are the current strategies employed to decrease suicide rate in BD. There are no current biologic variables that can help identify the BD individuals at risk for suicide. The identification of such variables would prompt a Copernical revolution in the treatment of these individuals. Suicide behavior is a complex phenotype. It is unreasonable to look for the “suicide gene” or “suicide genetic variation”. It is more likely that several genetic variations distributed in different genes concur in shaping the general risk for suicide behavior. Our paper enforces the complex, multigenetic background behind suicidal behavior, since the lack of significant association on single gene analysis, although we should also report some suggestive trends on AOPEP gene. In addition, environmental factors related to distal and proximal history, e.g., early-life adversity and psychiatric disorder also need to be taken in consideration when trying to evaluate the risk of suicide. In particular, including epigenetic and epigenomic modifications in individuals could represent an important step for future studies to include the effects of environment on genes’ expression. Despite this, we have highlighted how some specific biological processes, when altered can contribute to the risk of suicide more than others. We also evidenced slight differences between Suicide Attempters, Not Worth and Hurt classes, with the first more related to neuro-like processes: Neurotransmission, and Development (in particular GABA and NOTCH which were unique for this class). While others interestingly correlated with processes such as ECM organization and glycosylation processes. Growing evidence is available correlating these cascades to brain functions [91,92], suggesting that they may underlie the development and progression of neurologic alterations which can affect suicide risk. In the present research the combined effect of several genetic variations is analyzed in order to identify BD individuals at risk for suicide. A hypothesis—free analysis is conducted throughout the whole genome of each individual. Genes and their embedded variations are grouped in consistent molecular pathways across the whole genome and tested for “enrichment”. That means that the molecular pathways that contain more genetic variations significantly associated with suicide behavior than expected by chance are identified. The result of the present investigation helps in prompting further analysis of specific molecular pathways. This knowledge can be used both for the identification of BD that have a genetic predisposition to suicide behavior, and to the engineering of specific drugs able to tackle the molecular pathways at risk.

## Figures and Tables

**Table 1:** Main Previous Findings about the Genetics of Suicide Behavior in Affective Disorders.

References	Sample	Main Findings	Type of Study
[16]	79 individuals with bipolar I 30 individuals with bipolar II 86 healthy controls	No association between bipolar disorder and the *SERT* gene. No association was found between suicidal behavior and the *SERT* gene.	Candidate gene, cases and controls
[17]	67 individuals with depressive disorders 28 individuals with bipolar disorder 106 healthy controls	No association between the *5-HT2A* polymorphism *1438G/A* and the patient group or suicide attempts.	Candidate gene, cases and controls
[18]	46 individuals with depressive disorders 34 individuals with bipolar disorder 92 healthy controls	No association between the serotonin transporter polymorphism in *SLC6A4* gene and mood disorders or suicide attempts.	Candidate gene, cases and controls
[19]	70 individuals with a history of suicide attempts and various psychiatric disorders 42 individuals with MDD 10 individuals with bipolar disorder 97 healthy controls	No association between the *G2457A* polymorphism in *ABCG1* gene and affective disorders or suicidal behavior.	Candidate gene, cases and controls
[20]	2025 affected relative pairs with depressive disorders and mood disorders	Significant association between regions at 2p, 5q, 6q, 11q and Xq and suicide attempt. Strongest evidence for the phenotype Depression Spectrum Disorder was found at D8S1145 marker at 8p22-p21. Significant association between recurrent, early-onset major depressive disorder (RE-MDD) and Xq at DXS1047 marker. For all depressive phenotypes significant correlation with D8S1145 and suicide attempt.	Genome-wide linkage
[21]	9265 individuals, probands with alcohol dependence and biological relatives	Significant association for the phenotype “ever tried suicide” and chromosome 2 near D2S1790. Some association between the quantitative suicidality index and chromosome 1 near D1S1602, and chromosome 3 near D1S1602.	Genome-wide linkage
[22]	106 individuals with completed suicide and MDD or depression not otherwise specified 152 controls with MDD	The variants *5-HTTLPR* and *STin2* in *5-HTT* were considered. A significant association was found between suicide completion and having at least one copy of the *STin2* 10 allele. Added a positive family history of suicide risk increases the risk of suicide 5.56 times after adjustment for other clinical risk factors.	Candidate gene, cases and controls
[23]	1060 individuals with bipolar disorder from 154 multiplex families	Genome-wide significance between 6q25.2 at D6S2436 and suicidal behavior. Suggestive linkage was observed on 2q24.1 at D2S1353, 4p16.1 at D4S2366, 6q24.3 at D6S1848 and 10q25.3 at D10S1237.	Genome-wide linkage
[24]	162 individuals, multiplex bipolar pedigrees	Suggestive linkage signal between 2p12 and suicide attempt; from D2S1394 on 2p13 to D2S2972 on 2q11, including *TACR1* and *TGOLN2*. The second suggestive association was found at 6q26 at D6S1277.	Genome-wide linkage
[25]	154 individuals with MDD 154 healthy, age and gender matched controls	No association between the *Val66Met* polymorphism of the *BDNF* and development of MDD. Significant association between the dose of the Met allele and the clinical features psychotic and suicidal behavior, which suggest association with severe MDD.	Candidate gene, cases and controls
[26]	3117 individuals with bipolar disorder 1273 individuals with MDD	Suicide attempts in the bipolar sample were associated with following SNPs: rs1466846 (*TBL1XR1*), rs924134 (*IRX2*), rs6548036 (*CAPN13*), rs1457463 (*ZNF409*), rs11130703 (*FLJ42117*). Suicide attempts in the MDD sample were associated with following SNPs: rs2576377 (*ABI3BP*), rs2601098 (*SLC4A4*), rs1417259 (*LRRC44*), rs7655668 (*SLC4A4*), rs12462673 (*HAS1*), rs6737169 (*ARL6IP2*). None of these results were replicated. Modest support was found for candidate genes *FKBP5* and *NGFR* (*p75NTR*).	Genome-wide association study
[27]	2023 individuals with MDD	The quantitative SSU score showed suggested association for rs4751955 (*GFRA1*). For the discrete trait of serious suicidal attempts suggested association was found at rs203136 (*KIAA1244*). None of these results were replicated. Candidate gene analysis supported the association of a polymorphism in *NTRK2* with suicidality.	Genome-wide association study
[28]	2836 individuals with bipolar disorder	Associated SNP (rs300774) on 2p25 related to the *ACP1* gene was marginally associated with suicide risk.	Genome-wide association study
[29]	250 individuals with treatment resistant MDD	No association was found between genotyped SNPs in the *COMT* gene and suicide attempts and suicide risk. Significant association between suicide risk and non-responders to antidepressant treatment was found.	Candidate gene, genome-wide association study
[12]	4047 individuals with MDD, recurrent MDD, and bipolar disorder	Suggestive significance for suicide attempt and Rs935194. Meta-analysis found SNPs with suggestive significance: rs17173608 (*RARRES2*), rs17387100 (*PROM1*), rs3781878 (*NCAM1*), rs17010519 (*HK2*), rs13049531 (*RCAN1*), rs9394433 (*RNF8*). Polygenic scores for MDD significantly predicted suicidal ideation, this was also found for suicide attempt in a validation dataset.	Genome-wide association study, polygenic score analysis, meta-analysis.
[30]	959 individuals with bipolar disorder	Associated genes with suicide severity were found at chromosome 8q12 (*LINC000968/PENK*), and at chromosome 10p11.2 (*CCDC7/C10orf6/ITGB1*) Suggestive genes associated with suicide attempt were found at 8q12-q21 *(IL7*) and at 18q22 (*TMX3*).	Genome-wide association study, meta-analysis.
[31]	475 individuals, suicide attempters and suicides 1133 controls, with MDD or healthy	No association between suicidal behavior and CNV was found at genome-wide significant level. Highlighted results were CNVs at 6p22.2 including a H1 gene cluster and at 12q12 (*LRRK2*).	Cases and controls, PCR.
[13]	577 individuals, suicide attempters and suicides 1233 individuals, non-attempter psychiatric and healthy controls	Comparing suicidal behavior (SB) to no SB, no SNPs reached genome wide significance, five SNPs had significant levels; rs11852984 (intergenic), rs6480463 (*ADAMTS14*), rs4575 (*PSME2/RNF31*), rs336284 (*TBX20*) and rs3019286 (*STK3*). Pathway analysis identified: “Cellular assembly and organization”, “nervous system development and function”, “cell death and survival”, “immunological disease”, “infectious disease” and “inflammatory response”.	Genome-wide association study, pathway analysis
[32]	660 individuals with severe suicide attempt 88 individuals with SCZ-related diagnoses 85 individuals with MDD 489 healthy individuals	The top polygenes associated with neurodevelopment and suicide attempt were: *CDH4*, *CDH12*, *CDH11*, *CDH13*, *CDH20*, *NRXN1*, *NRXN3*, *FGF12*, *NELL1*, *EPHB2*, *EPHA6*, *GLI2*, *MIXL1*, *MAML2*, *MS12*, *NTRK3*, *NPAS3*, *ODZ4*, *MYCBP2*. Support evidence of a polygenic neurodevelopmental etiology in SB, also in absence of major psychiatric diagnoses.	Genome-wide association study, polygenic risk scores
[11]	GWAS: 473 individuals, cases 9778 individuals, controls Including psychiatric disorders and suicide attempters Clinical case-control: 51 individuals, suicide attempters 112 controls	Meta-analysis found significant association between suicide attempt and a locus on chromosome 6, near *MRAP2* and *CEP162*, this consisted of 12 SNPs, peak SNP rs12524136-T, this was replicated in a meta-analysis of all studies and ancestral subgroups. Suggestive association was found for suicide attempt and bipolar disorder regarding the polygenic risk scores.	Genome-wide association study, meta-analysis, cases and controls
[33]	1780 individuals with schizophrenia 1768 healthy matched controls	A 10 times higher mortality rate as well as high risk of multiple suicide attempts was replicated for persons with schizophrenia compared to the controls. No genetic overlap was found between PRS and mortality, or between PRS and multiple suicide attempts. Family history of mental disorders was found to be associated with higher mortality and multiple suicide attempts.	Cases and controls, polygenic risk scores
[9]	6569 individuals with psychiatric disorders, all suicide attempters 17232 individuals with psychiatric disorders, all non-attempters	Three significant loci were found: for MDD a SNP rs45593736 (an intron of the *ARL5B*), for BD an insertion-deletion polymorphism *chr4_23273116_D* (an intronic variant in the noncoding RNA *LOC105374524*). Polygenic risk scores for MDD were significantly associated with suicide attempt in MDD (R2 = 0.25%), BD (R2 = 0.24%) and schizophrenia (R2 = 0.40%).	Genome-wide association study, polygenic risk scores
[34]	6320 individuals with psychiatric disorders and SUD.	One genome-wide significant SNP s1677091 (*LDHB*). Other associations were rs683813 (*ARNTL2*), rs72740082 (*FAH*) and s11876255. Significant genetic overlap between MDD and suicide attempt severity was estimated up to 0.7% using PRS.	Genome-wide association study, polygenic risk scores
[10]	2433 individuals, all attempters, including psychiatric disorders 334766 controls 61676 individuals from electronic health records	For suicide attempt significant heritability from common variation was estimated to 4%, and significant genetic correlation was found for depressive symptoms, neuroticism, MDD, schizophrenia and insomnia. For one sample two genomic regions with genome-wide significance were identified on chromosomes 5 and 19, the most significant SNPs being rs12972617 and rs12972618.	Genome-wide association study, polygenic risk scores, machine learning
[14]	6024 individuals, all attempters, including psychiatric disorders 44240 controls, non-attempters, including psychiatric disorders	Suggestive associations between SNPs, rs6880062 and rs6880461, and suicide attempt. Adjusted for mental disorders three significant associations were found on chromosome 20; rs4809706, rs4810824 and rs6019297. Heritability was found to be 4.6%, adjusted for mental disorders heritability was 1.9%.	Genome-wide association study

**Table 2:** Test between group IBS (identical by state) differences for stratification factors.

	Not_Worth	Fantasies about Hurting Suicide	Tried Suicide
T1: Case/control less similar	*p* = 0.209928	*p* = 0.209928	*p* = 0.553834
T2: Case/control more similar	*p* = 0.790082	*p* = 0.790082	*p* = 0.446176
T3: Case/case less similar than control/control	*p* = 0.207738	*p* = 0.207738	*p* = 0.553984
T4: Case/case more similar than control/control	*p* = 0.792272	*p* = 0.792272	*p* = 0.446026
T5: Case/case less similar	*p* = 0.200218	*p* = 0.200218	*p* = 0.563414
T6: Case/case more similar	*p* = 0.799792	*p* = 0.799792	*p* = 0.436596
T7: Control/control less similar	*p* = 0.791442	*p* = 0.791442	*p* = 0.446156
T8: Control/control more similar	*p* = 0.208568	*p* = 0.208568	*p* = 0.553854
T9: Case/case less similar than case/control	*p* = 0.788322	*p* = 0.788322	*p* = 0.446346
T10: Case/case more similar than case/control	*p* = 0.211688	*p* = 0.211688	*p* = 0.553664
T11: Control/control less similar than case/control	*p* = 0.790802	*p* = 0.790802	*p* = 0.446166
T12: Control/control more similar than case/control	*p* = 0.209208	*p* = 0.209208	*p* = 0.553844

**Table 3:** Sample characteristics and clinical covariate analysis.

Variable		Not Worth Class (Yes, No)	Hurt Class (Yes, No)	Suicide Attempters Class (Yes, No)
**Age**				
mean: 41.69 +/− 12.26		Yes: 41.1 +/− 11.49	Yes: 39.85 +/− 11.14	Yes: 35.28 +/− 11.24
		No: 42.24 +/− 12.9	No: 42.45 +/− 12.62	No: 41.98 +/− 12.22
		t = 1.5936, df = 1153.9, *p*-value = 0.111	t = 3.4719, df = 706.97, *p*-value = 0.0005	t = 4.1073, df = 54.366, *p*-value = 0.0001
Gender				
Males = 670 (58.01%)	Females = 485 (41.99%)	X-squared = 0.19584, df = 1, *p*-value = 0.6581	X-squared = 0.74851, df = 1, *p*-value = 0.3869	X-squared = 0.37699, df = 1, *p*-value = 0.5392
Race				
Asian or Pacific Islander *n* = 25 (2.13%)	No Primary Race *n* = 6 (0.51%)	**X-squared = 12.689, df = 6,** ***p*-value = 0.04826**	X-squared = 11.531, df = 6, *p*-value = 0.07328	X-squared = 1.9257, df = 6, *p*-value = 0.9264
Black or African American *n* = 53 (4.51%)	Other, Specify *n* = 8 (0.68%)			
Native American, Eskimo or Aleut *n* = 5 (0.43%)	N/A *n* = 17 (1.45%)			
White or Caucasian *n* = 1060 (90.29%)				
Marital status				
Divorced *n* = 234 (19.93%)	Separated/No longer living as married *n* = 56 (4.77%)	**X-squared = 16.528, df = 6,** ***p*-value = 0.01118**	**X-squared = 17.963, df = 6,** ***p*-value = 0.006327**	X-squared = 11.739, df = 6, *p*-value = 0.06806
Living as Married *n* = 28 (2.39%)	Widowed *n* = 15 (1.28%)			
Married *n* = 435 (37.05%)	Unknown *n* = 17 (1.45%)			
Never Married (never lived as) *n* = 389 (33.13%)				
Living alone				
Yes *n* = 297 (25.3%)	Unknown *n* = 16 (1.36%)	**X-squared = 11.023, df = 2,** ***p*-value = 0.00404**	X-squared = 2.4716, df = 2, *p*-value = 0.2906	X-squared = 1.6656, df = 2, *p*-value = 0.4348
No *n* = 861 (73.34%)				
Less than seventh grade *n* = 0 (0%)	College Diploma (Bachelors Degree) *n* = 342 (29.13%)	X-squared = 13.408, df = 7, *p*-value = 0.06278	X-squared = 6.6033, df = 7, *p*-value = 0.4713	X-squared = 14.007, df = 7, *p*-value = 0.05105
Seventh grade–ninth grade *n* = 7 (0.6%)	Technical School or Associates Degree *n* = 131 (11.16%)			
Partial High School *n* = 17 (1.45%)	Graduate or Professional Degree *n* = 219 (18.65%)			
High School Diploma or GED *n* = 156 (13.29%)	Unknown *n* = 17 (1.45%)			
Some college (at least one year) *n* = 285 (24.28%)				
Job				
Clerical and sales workers *n* = 237 (20.19%)	Professional *n* = 349 (29.73%)	**X-squared = 12.654, df = 6,** ***p*-value = 0.04888**	**X-squared = 13.007, df = 6,** ***p*-value = 0.04292**	X-squared = 11.523, df = 6, *p*-value = 0.07349
Craftsmen and kindred workers *n* = 135 (11.5%)	Other *n* = 170 (14.48%)			
Laborers, operatives and kindred workers *n* = 91 (7.75%)	Unknown *n* = 40 (3.41%)			
Managers and administrators *n* = 152 (12.95%)				
Employment				
Disabled *n* = 230 (19.59%)	Part-time for pay *n* = 164 (13.97%)	**X-squared = 31.957, df = 8,** ***p*-value = 9.48 × 10^−5^**	X-squared = 14.495, df = 8, *p*-value = 0.06974	X-squared = 13.29, df = 8, *p*-value = 0.1022
Full-time *n* = 358 (30.49%)	Retired *n* = 46 (3.92%)			
Homemaker *n* = 56 (4.77%)	Unemployed *n* = 259 (22.06%)			
Leave of Absence *n* = 22 (1.87%)	Unknown *n* = 22 (1.87%)			
Other *n* = 17 (1.45%)				
Earnings				
less than $10,000 *n* = 565 (48.13%)	$75,000–$99,999 *n* = 31 (2.64%)	X-squared = 18.28, df = 10, *p*-value = 0.05043	X-squared = 5.8668, df = 10, *p*-value = 0.8263	X-squared = 6.8626, df = 10, *p*-value = 0.7384
$10,000–$19,999 *n* = 141 (12.01%)	$100,000–$149,999 *n* = 17 (1.45%)			
$20,000–$29,999 *n* = 110 (9.37%)	$150,000 or more *n* = 18 (1.53%)			
$30,000–$39,999 *n* = 96 (8.18%)	Refused *n* = 3 (0.26%)			
$40,000–$49,999 *n* = 63 (5.37%)	Unknown *n* = 40 (3.41%)			
$50,000–$74,999 *n* = 90 (7.67%)				
Home income				
less than $10,000 *n* = 166 (14.14%)	$75,000–$99,999 *n* = 107 (9.11%)	**X-squared = 33.938, df = 11,** ***p*-value = 0.0003702**	X-squared = 15.01, df = 11, *p*-value = 0.1821	X-squared = 18.084, df = 11, *p*-value = 0.07966
$10,000–$19,999 *n* = 149 (12.69%)	$100,000–$149,999 *n* = 117 (9.97%)			
$20,000–$29,999 *n* = 125 (10.65%)	$150,000–$199,999 *n* = 40 (3.41%)			
$30,000–$39,999 *n* = 95 (8.09%)	$200,000 or more *n* = 37 (3.15%)			
$40,000–$49,999 *n* = 86 (7.33%)	Refused *n* = 11 (0.94%)			
$50,000–$74,999 *n* = 150 (12.78%)	Unknown *n* = 91 (7.75%)			
Personal income				
less than $10,000 *n* = 454 (38.67%)	$75,000–$99,999 *n* = 36 (3.07%)	X-squared = 17.176, df = 10, *p*-value = 0.07057	X-squared = 8.5879, df = 10, *p*-value = 0.5716	X-squared = 10.109, df = 10, *p*-value = 0.431
$10,000–$19,999 *n* = 199 (16.95%)	$100,000–$149,999 *n* = 32 (2.73%)			
$20,000–$29,999 *n* = 122 (10.39%)	$150,000 or more *n* = 25 (2.13%)			
$30,000–$39,999 *n* = 106 (9.03%)	Refused *n* = 4 (0.34%)			
$40,000–$49,999 *n* = 69 (5.88%)	Unknown *n* = 43 (3.66%)			
$50,000–$74,999 *n* = 84 (7.16%)				
Medical insurance				
Yes *n* = 955 (81.35%)	Unknown *n* = 23 (1.96%)	**X-squared = 7.4714, df = 2,** ***p*-value = 0.02386**	X-squared = 0.35551, df = 2, *p*-value = 0.8371	X-squared = 0.32662, df = 2, *p*-value = 0.8493
No *n* = 196 (16.7%)				
Limited mental care				
Yes *n* = 469 (39.95%)	N/A *n* = 448 (38.16%)	**X-squared = 23.657, df = 3,** ***p*-value = 2.945 × 10^−5^**	X-squared = 3.0221, df = 3, *p*-value = 0.3882	X-squared = 3.6797, df = 3, *p*-value = 0.2982
No *n* = 167 (14.22%)	Unknown *n* = 90 (7.67%)			
inpatient day per year (Number of days admitted in the hospital as inpatients)				
Yes *n* = 351 (29.9%)	N/A *n* = 705 (60.05%)	**X-squared = 16.111, df = 3,** ***p*-value = 0.001076**	X-squared = 3.9117, df = 3, *p*-value = 0.2712	X-squared = 2.7955, df = 3, *p*-value = 0.4242
No *n* = 72 (6.13%)	Unknown *n* = 46 (3.92%)			
outpatient day per year (Number of days admitted in the hospital as outpatients)				
Yes *n* = 398 (33.9%)	N/A *n* = 705 (60.05%)	**X-squared = 13.383, df = 3,** ***p*-value = 0.003878**	X-squared = 2.3971, df = 3, *p*-value = 0.4942	X-squared = 2.7176, df = 3, *p*-value = 0.4372
No *n* = 31 (2.64%)	Unknown *n* = 40 (3.41%)			
Life not worth living				
Yes *n* = 567	No *n* = 607	/	/	/
Fantasies on a violent suicide				
Yes *n* = 347	No *n* = 827	/	/	/
Attempted suicide				
Yes *n* = 51	No *n* = 1123	/	/	/

Bold: It identifies the significant association, it is used to navigate the table in a faster way.

**Table 4:** Splicing Analysis of rs2767403.

Overview (on GRCh38.13)			
**Mutation**			**Gene**
Name:	rs2767403	9:94811838 C/G	C9orf3 (AOPEP)
HGVS Nomenclature:	ENST00000277198.6:c.1364+10836C > G		9:94726669-95148264
Analysis Results			
Signal	**Interpretation**		
New Donor splice site	Activation of a cryptic Donor site. Potential alteration of splicing		
Details			
**Name**	**Position**	**Sequences**	**Variation**
HSF Donor site (matrix GT)	chr9:94811835	TCTCTCTGA > TCTGTCTGA	38.58 > 65.72 (70.35%)
Analysis in silico was performed with Genomnis Human Splicing Finder software (www.genomnis.com/, accessed on 21 April 2021)			

**Table 5:** Result from the molecular pathway analysis (main analysis).

Attempted				Not Worth			
ID	Description	*p*.adjust	*q* Value	ID	Description	*p*.adjust	*q* Value
R-HSA-112316	Neuronal System	5.07 × 10^−6^	4.83 × 10^−6^	R-HSA-5576891	Cardiac conduction	1.04 × 10^−5^	9.97 × 10^−6^
R-HSA-112314	Neurotransmitter receptors and postsynaptic signal transmission	3.78 × 10^−4^	3.60 × 10^−4^	R-HSA-373752	Netrin-1 signaling	2.09 × 10^−5^	2.01 × 10^−5^
R-HSA-112315	Transmission across Chemical Synapses	6.87 × 10^−4^	6.55 × 10^−4^	R-HSA-428542	Regulation of commissural axon pathfinding by SLIT and ROBO	3.17 × 10^−5^	3.04 × 10^−5^
R-HSA-977443	GABA receptor activation	1.46 × 10^−2^	1.39 × 10^−2^	R-HSA-112316	Neuronal System	4.16 × 10^−5^	3.99 × 10^−5^
R-HSA-350054	NOTCH-HLH transcription pathway	1.84 × 10^−2^	1.76 × 10^−2^	R-HSA-445095	Interaction between L1 and Ankyrins	8.71 × 10^−5^	8.35 × 10^−5^
R-HSA-373752	Netrin-1 signaling	2.50 × 10^−2^	2.38 × 10^−2^	R-HSA-397014	Muscle contraction	9.81 × 10^−5^	9.41 × 10^−5^
R-HSA-446728	Cell junction organization	2.70 × 10^−2^	2.58 × 10^−2^	R-HSA-373760	L1CAM interactions	1.50 × 10^−4^	1.43 × 10^−4^
R-HSA-1500931	Cell-Cell communication	3.27 × 10^−2^	3.12 × 10^−2^	R-HSA-5083635	Defective B3GALTL causes Peters-plus syndrome (PpS)	5.91 × 10^−4^	5.67 × 10^−4^
R-HSA-428542	Regulation of commissural axon pathfinding by SLIT and ROBO	3.75 × 10^−2^	3.57 × 10^−2^	R-HSA-5173214	O-glycosylation of TSR domain-containing proteins	7.40 × 10^−4^	7.09 × 10^−4^
R-HSA-418990	Adherens junctions interactions	3.75 × 10^−2^	3.57 × 10^−2^	R-HSA-1650814	Collagen biosynthesis and modifying enzymes	2.40 × 10^−3^	2.30 × 10^−3^
R-HSA-421270	Cell-cell junction organization	4.29 × 10^−2^	4.10 × 10^−2^	R-HSA-1474244	Extracellular matrix organization	2.81 × 10^−3^	2.69 × 10^−3^
**Hurt**				R-HSA-8948216	Collagen chain trimerization	3.18 × 10^−3^	3.05 × 10^−3^
**ID**	**Description**	***p*.adjust**	***q* Value**	R-HSA-375165	NCAM signaling for neurite out-growth	3.18 × 10^−3^	3.05 × 10^−3^
R-HSA-112316	Neuronal System	2.23 × 10^−7^	2.11 × 10^−7^	R-HSA-983712	Ion channel transport	3.82 × 10^−3^	3.66 × 10^−3^
R-HSA-5576891	Cardiac conduction	2.23 × 10^−7^	2.11 × 10^−7^	R-HSA-3000178	ECM proteoglycans	8.98 × 10^−3^	8.61 × 10^−3^
R-HSA-397014	Muscle contraction	2.49 × 10^−6^	2.36 × 10^−6^	R-HSA-2022928	HS-GAG biosynthesis	9.01 × 10^−3^	8.64 × 10^−3^
R-HSA-112315	Transmission across Chemical Synapses	4.62 × 10^−4^	4.37 × 10^−4^	R-HSA-5578775	Ion homeostasis	9.01 × 10^−3^	8.64 × 10^−3^
R-HSA-373760	L1CAM interactions	4.62 × 10^−4^	4.37 × 10^−4^	R-HSA-936837	Ion transport by P-type ATPases	1.04 × 10^−2^	1.00 × 10^−2^
R-HSA-445095	Interaction between L1 and Ankyrins	8.83 × 10^−4^	8.37 × 10^−4^	R-HSA-5173105	O-linked glycosylation	1.49 × 10^−2^	1.43 × 10^−2^
R-HSA-112314	Neurotransmitter receptors and postsynaptic signal transmission	1.60 × 10^−3^	1.52 × 10^−3^	R-HSA-5576892	Phase 0—rapid depolarization	1.71 × 10^−2^	1.64 × 10^−2^
R-HSA-5576892	Phase 0—rapid depolarization	2.02 × 10^−3^	1.92 × 10^−3^	R-HSA-3906995	Diseases associated with O-glycosylation of proteins	1.89 × 10^−2^	1.81 × 10^−2^
R-HSA-1474244	Extracellular matrix organization	4.49 × 10^−3^	4.26 × 10^−3^	R-HSA-112315	Transmission across Chemical Synapses	1.90 × 10^−2^	1.82 × 10^−2^
R-HSA-5578775	Ion homeostasis	5.64 × 10^−3^	5.34 × 10^−3^	R-HSA-419037	NCAM1 interactions	2.28 × 10^−2^	2.18 × 10^−2^
R-HSA-373752	Netrin-1 signaling	5.64 × 10^−3^	5.34 × 10^−3^	R-HSA-1474290	Collagen formation	4.63 × 10^−2^	4.44 × 10^−2^
R-HSA-5173105	O-linked glycosylation	4.12 × 10^−2^	3.90 × 10^−2^				

**Table 6:** Result from the molecular pathway analysis (exploratory analysis).

ID	Description	*p*.adjust	*q* Value
R-HSA-112316	Neuronal System	3.94 × 10^−4^	3.67 × 10^−4^
R-HSA-9013508	NOTCH3 Intracellular Domain Regulates Transcription	5.05 × 10^−3^	4.71 × 10^−3^
R-HSA-210744	Regulation of gene expression in late stage (branching morphogenesis) pancreatic bud precursor cells	5.05 × 10^−3^	4.71 × 10^−3^
R-HSA-350054	NOTCH-HLH transcription pathway	5.82 × 10^−3^	5.42 × 10^−3^
R-HSA-5173105	O-linked glycosylation	5.95 × 10^−3^	5.54 × 10^−3^
R-HSA-8941856	RUNX3 regulates NOTCH signaling	2.69 × 10^−2^	2.50 × 10^−2^
R-HSA-186712	Regulation of β-cell development	3.11 × 10^−2^	2.89 × 10^−2^
R-HSA-373760	L1CAM interactions	3.29 × 10^−2^	3.06 × 10^−2^
R-HSA-112315	Transmission across Chemical Synapses	3.36 × 10^−2^	3.13 × 10^−2^
R-HSA-445095	Interaction between L1 and Ankyrins	4.48 × 10^−2^	4.17 × 10^−2^
R-HSA-2122947	NOTCH1 Intracellular Domain Regulates Transcription	4.48 × 10^−2^	4.17 × 10^−2^
R-HSA-163685	Integration of energy metolism	4.48 × 10^−2^	4.17 × 10^−2^
R-HSA-9012852	Signaling by NOTCH3	4.48 × 10^−2^	4.17 × 10^−2^
R-HSA-5576892	Phase 0—rapid depolarisation	4.68 × 10^−2^	4.35 × 10^−2^
R-HSA-9013695	NOTCH4 Intracellular Domain Regulates Transcription	4.68 × 10^−2^	4.35 × 10^−2^

## Data Availability

The Data and biomaterials analyzed in this study were obtained from the STEP-BD study (2N01MH080001-001), available on NIMH Repository with permission of NIMH (https://www.nimhgenetics.org/, accessed on 21 April 2021).

## Appendix A. Definitions of Suicide—Related Phenotypes

The term suicidal ideation, i.e., suicidal thoughts or ideas, describes a broad range of contemplations, wishes and preoccupations with death and suicide. There is no established definition of suicidal ideation, making the clinical assessment difficult, and also research is hindered by the need of operational definitions leading to inability to compare findings [117].

Suicide attempt is defined by WHO as “any non-fatal suicidal behavior”, further described as any “intentional self-inflicted poisoning, injury or self-harm which may or may not have a fatal intent or outcome”, while suicide is defined as “the act of deliberately killing oneself”. Worldwide approximately 800,000 individuals commit suicide each year, it is the 10th leading cause of death overall and the number is probably underestimated due to the sensitivity of the circumstance and countries where suicide is illegal.

## Appendix B. Clinical Risk Factors and Prevention Measures for Suicide Behavior in BD

Risk factors—Some risk factors are seen as general risk factors for suicide, especially regarding socio-demographic factors; gender (male), marital status (single, widowed, divorced), living alone, age under 35 years or over 75 years, no children and unemployment. In addition, some clinical risk factors are overall risk factors for committing suicide, e.g., history of suicide attempt and family history of completed suicide. Other clinical risk factors are related to the characteristics and phase of the illness: predominant depressive episode, major depressive episode, rapid cycling subtype, earlier age of onset, longer duration of untreated illness, previous hospitalization, concurrent medical comorbidity and mood-incongruent psychotic symptoms. Psychiatric comorbidity in the form of personality disorders (borderline, antisocial, histrionic and narcissistic) also higher the risk.

Prevention strategies—When it comes to the prevention of suicide in BD, several studies have shown the anti-suicidal effect of lithium treatment, including a large Swedish study with over 50,000 patients. The register-based longitudinal study found a decreased incidence of suicide-related events of 14% compared to those that received valproate [118]. Another study found a 5-fold greater risk of suicide attempts among patients with poor adherence to long-term lithium maintenance treatment compared to those with high adherence [119]. Electroconvulsive therapy (ECT) has proven to be effective in treating acute suicidal danger in severely depressed patients; furthermore ECT also prevents subsequent suicidal behavior [120]. Medical treatment should never stand alone; a general national guideline for preventing suicide could consist of education of the patient as well as friends and family, a 24-h telephone hotline, the development of a personalized safety planning and for some psychotherapy.
